# Applying and refining DNA analysis to determine the identity of plant material extracted from the digestive tracts of katydids

**DOI:** 10.7717/peerj.6808

**Published:** 2019-05-03

**Authors:** Laurel B. Symes, Nicole L. Wershoven, Lars-Olaf Hoeger, Jessica S. Ralston, Sharon J. Martinson, Hannah M. ter Hofstede, Christine M. Palmer

**Affiliations:** 1 Bioacoustics Research Program, Lab of Ornithology, Cornell University, Ithaca, NY, USA; 2 Department of Biological Sciences, Dartmouth College, Hanover, NH, USA; 3 Natural Sciences Department, Castleton University, Castleton, VT, USA

**Keywords:** Diet, Foraging, Tettigoniidae, Gut contents, DNA sequencing

## Abstract

**Background:**

Feeding habits are central to animal ecology, but it is often difficult to characterize the diet of organisms that are arboreal, nocturnal, rare, or highly mobile. Genetic analysis of gut contents is a promising approach for expanding our understanding of animal feeding habits. Here, we adapt a laboratory protocol for extracting and sequencing plant material from gut contents and apply it to Neotropical forest katydids (Orthoptera: Tettigoniidae) on Barro Colorado Island (BCI) in Panama.

**Methods:**

Our approach uses three chloroplast primer sets that were previously developed to identify vegetation on BCI. We describe the utility and success rate of each primer set. We then test whether there is a significant difference in the amplification and sequencing success of gut contents based on the size or sex of the katydid, the time of day that it was caught, and the color of the extracted gut contents.

**Results:**

We find that there is a significant difference in sequencing success as a function of gut color. When extracts were yellow, green, or colorless the likelihood of successfully amplifying DNA ranged from ~30–60%. When gut extracts were red, orange, or brown, amplification success was exceptionally low (0–8%). Amplification success was also higher for smaller katydids and tended to be more successful in katydids that were captured earlier in the night. Strength of the amplified product was indicative of the likelihood of sequencing success, with strong bands having a high likelihood of success. By anticipating which samples are most likely to succeed, we provide information useful for estimating the number of katydids that need to be collected and minimizing the costs of purifying, amplifying, and sequencing samples that are unlikely to succeed. This approach makes it possible to understand the herbivory patterns of these trophically important katydids and can be applied more broadly to understand the diet of other tropical herbivores.

## Introduction

**Figure 1:**
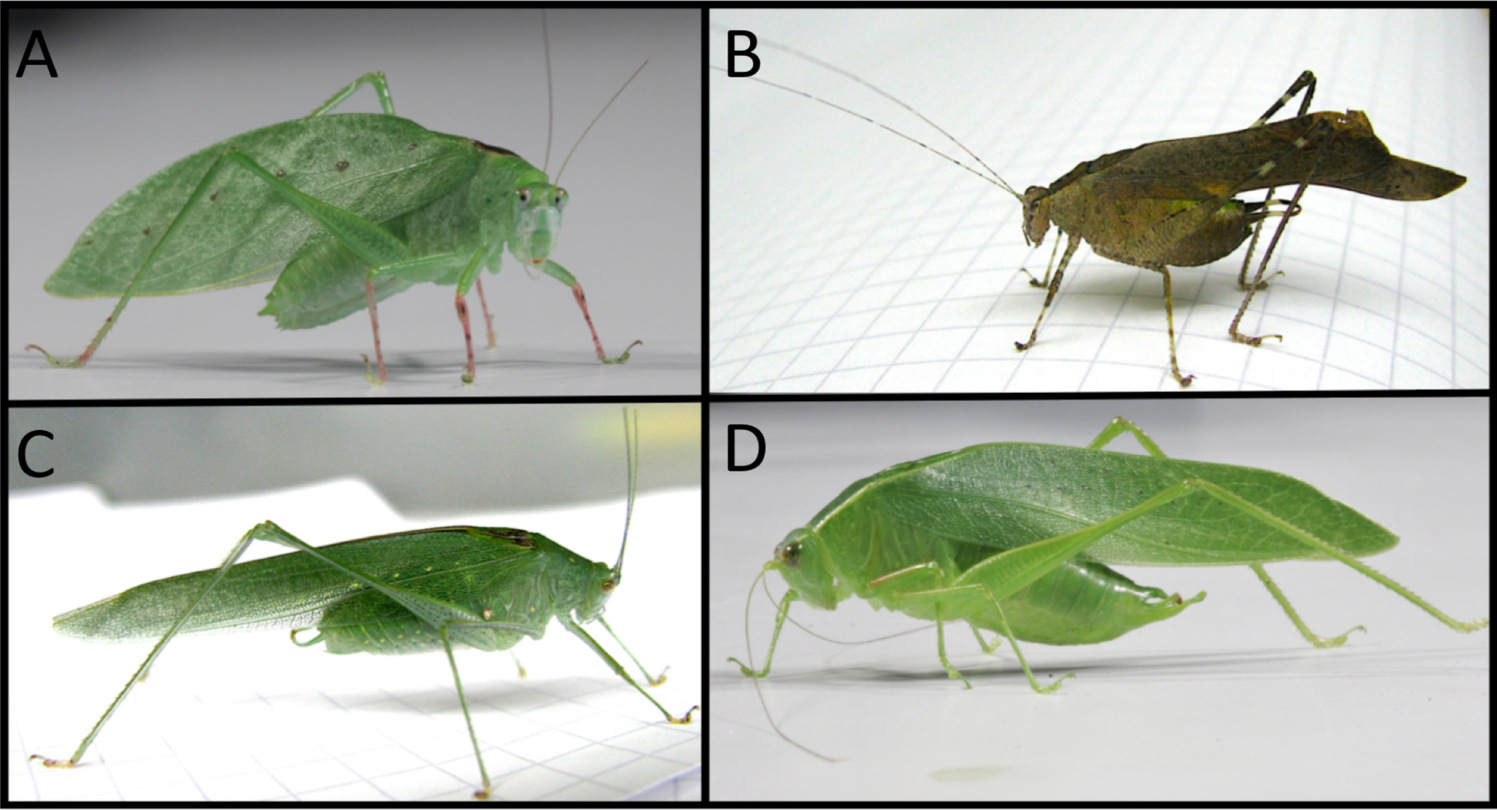
Four species of Phaneropterine katydids from Barro Colorado Island. (A) *Phylloptera dimidiata*, (B) *Dolichocercus latipennis*, (C) *Anaulacomera spatulata*, (D) *Chloroscirtus discocercus*. Photos by Hannah M. ter Hofstede and Laurel B. Symes.

The diet of organisms varies both within and between species and is intricately connected with distribution patterns, energy availability, metabolic processes, and predation risk (Scriber & Dowell, 1991; Tewfik et al., 2016; Glaser, Waechter & Bransome, 2015). Diet can be characterized using direct observation, but observation can be challenging in animals that are highly mobile, nocturnal, or feed in trees and other locations where it is difficult to make direct observations of foraging behavior (Barnett et al., 2010; Baker, Buckland & Sheaves, 2014). An alternative approach for characterizing diet is to examine gut contents (Ward, 1970). For herbivores, the effectiveness of gut content analysis can be greatly enhanced by extracting and sequencing plant DNA from the gut contents (Avanesyan, 2014; Matheson et al., 2008). Molecular analysis is particularly valuable in places like the tropics where plant diversity is exceptionally high and many rare diet components could be overlooked (Kricher, 1999; Buck, 1979). Previous researchers have used a single primer set to detect and amplify gut contents in insect feeding trials up to 22 h after the insect ingested the plant (Avanesyan, 2014). DNA sequencing demonstrated that the PCR product accurately reflected the plant used in the feeding trials (Avanesyan, 2014). Here, we adapt a method for the molecular analysis of gut contents for use in herbivorous tropical insects and provide insights on how to maximize the success and efficiency of the method (Avanesyan, 2014). Specifically, we translate the method to the field to assess the gut contents of Neotropical forest katydids on Barro Colorado Island (BCI) in Panama. This well-studied location has high biodiversity with more than 80 katydid species and more than 300 species of woody plants that serve as potential food sources (Nickle, 1992; Kress et al., 2009) (Fig. 1).

Katydids are abundant, diverse, and fill a central role in tropical forest food webs, both as major consumers of plant material and as a protein-rich food source for birds, bats, primates, reptiles, amphibians, and other invertebrates (Kalka & Kalko, 2006; Kalka, Smith & Kalko, 2008; ter Hofstede, Kalko & Fullard, 2010). Until recently, it has been nearly impossible to determine the dietary habits of these katydids because of their arboreal and nocturnal tendencies and because of the rich diversity of plant species that would need to be used in feeding trials (Lang et al., 2006; Croat, 1978). Molecular diet analysis by PCR amplification and Sanger sequencing allows for time- and cost-effective identification of consumed plants and has been successful in previous systems for identifying plants to genus and species in species-rich environments (Jurado-Rivera et al., 2008; García-Robledo et al., 2013). This method is particularly suited to field researchers seeking to understand what plants are being consumed across a large number of individuals because the method is relatively user-friendly and can be easily employed at field research stations without extensive expertise or additional resources. While next-generation sequencing (NGS) is more suitable to studies that seek to provide comprehensive information, this study seeks to present a method that is highly accessible to the average field researcher.

To characterize the katydid diet, we utilized three primer sets that were used previously to identify vegetation on BCI and that would have comparatively high efficacy in other Neotropical environments (Kress et al., 2009). Sequencing plant DNA from insect guts raises some unique additional challenges. Unlike plant material sampled directly from plants, material collected from insect guts will be at different stages of digestion, will be intermixed with various digestive compounds, and may differ substantially in chemical composition from the original plant material, affecting DNA binding and the chemistry of purification. In addition, insects in natural environments consume a variety of plant tissue including leaves, fruits, and seeds. These diverse inputs may affect the composition of the gut material and the concentration of compounds that could inhibit extraction. All of these variables can result in lower PCR amplification success than direct sampling of plant leaf material (Bafeel et al., 2012; Burgess et al., 2011). However, there are a number of factors that may be useful for anticipating and maximizing amplification success. First, feeding activity in katydids might be more common at certain times of night, and the amount of time the plant material that is in the guts might affect the sequencing success. Therefore, we predict that the time of night at which katydids are captured could influence sequencing success. Second, we test whether the success of gut content amplification is associated with the sex or size of the katydid as both of these factors might affect energetic demand, feeding frequency and location, and gut transit time. Third, the color and consistency of material in the guts might reveal what or how recently a katydid has eaten. Finally, we provide quantitative data on the probability of success in PCR and sequencing to make it easier to estimate how many insects should be collected. Accurate estimates are particularly important for researchers conducting field-based studies, where there is often a short window of time in which to obtain data and no local option for sequencing. Data on sequencing success are also useful for determining which samples are worth the cost of sequencing. Our data provide a guideline for field-based researchers seeking to maximize their efficiency and reduce their costs to answer population-level questions about insect diets.

## Materials and Methods

### Insect collection

Katydids (Orthoptera: Tettigoniidae) were collected by hand from lights around the field station on BCI, Panama (9.16335°, −79.8357°). Collections were made twice per night in January 2016 and January–March 2017, with one round of sampling between 23:00 and 01:00 (“midnight sample”) and a second round of sampling between 04:30 and 06:30 (“pre-dawn sample”). Katydids were frozen at −20 °C within 1 h of collection to inactive digestive enzymes.

### Dissection

**Figure 2:**
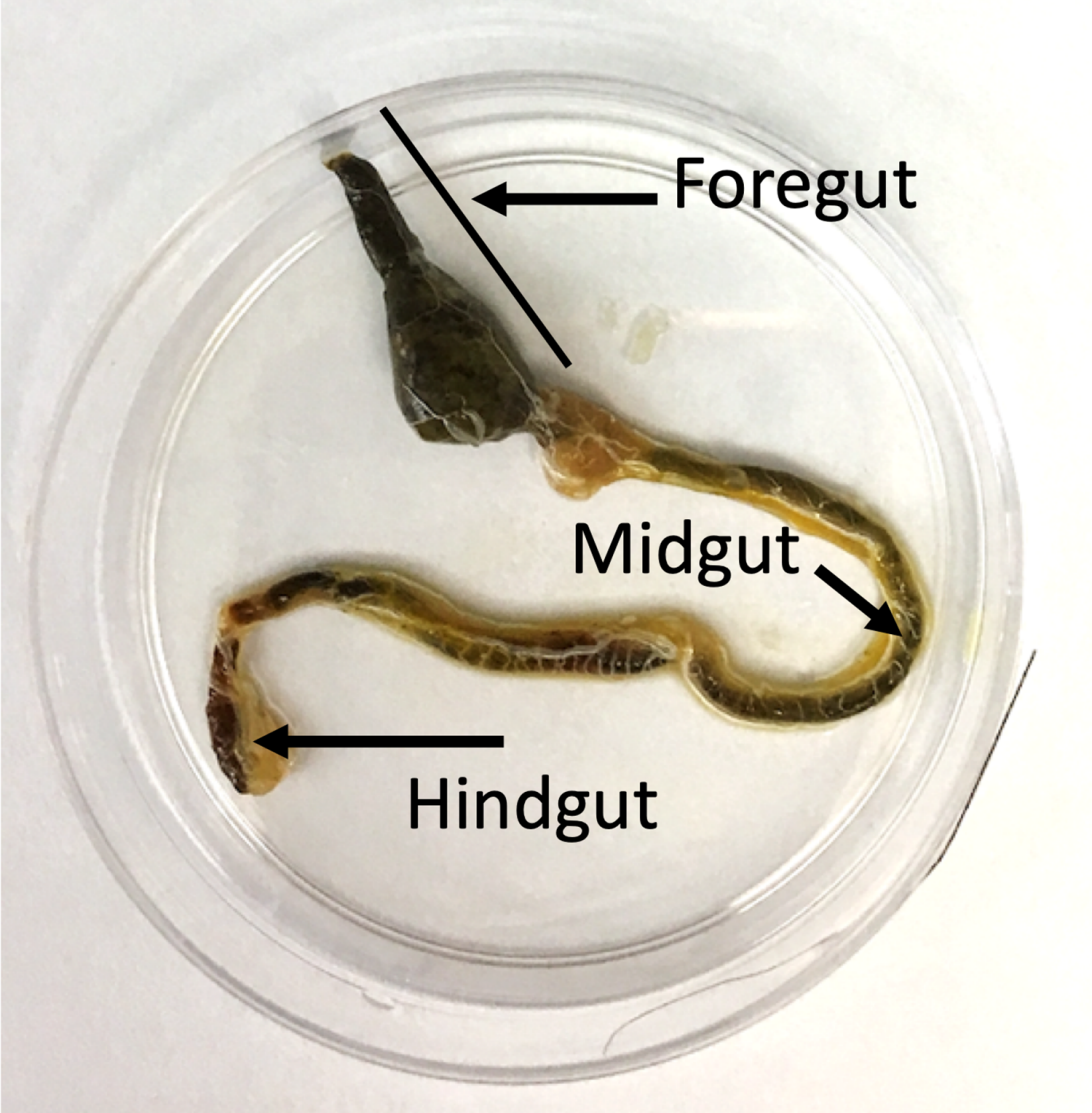
Internal anatomy of the katydid digestive system. The leaf material in the foregut of this katydid appears dark green while the material in the mid- and hindgut appears brown.

Prior to dissection, katydids were removed from the freezer and allowed to thaw briefly until the body was no longer frozen. At this time, the mass of the katydid was determined by using an American Weigh Scale (Gemini-20, Cumming, GA, USA). For each insect, the body cavity was cut shallowly along the ventral axis from below the head through the entirety of the thorax and abdomen to expose the digestive tract as previously described (Avanesyan, 2014). By pulling gently on the head, the entire digestive tract could be removed intact. The head was removed and the digestive tract was processed for further analysis (Fig. 2).

### DNA extraction and purification

Dissected guts were homogenized by hand using pellet pestles. DNA was purified following the manufacturer’s instructions using the QIAGEN DNeasy Plant Mini Kit (Qiagen 69104; Qiagen, Hilden, Germany) with the following modification. After initial homogenization, 50 µl of AP1 buffer was added to the tissue and the sample was crushed again. 350 µl of AP1 buffer was then added and the color of the suspended gut contents was determined visually by assigning color to one of eight categories. See Fig. S1 for examples of each color. All subsequent steps for DNA purification were as designated by the manufacturer. Samples were stored at −20 °C until processing through PCR to minimize DNase activity and maximize DNA integrity.

### PCR amplification and sequence analysis

Primers were utilized to amplify three conserved regions of the plastid genome following the procedure described in Kress et al. (2009) (Table 1). Of the three regions, the *rbcLa* region has the highest sequence conservation across plant species, the *psbA-trnH* region is intermediate, and the *matK* region is the most variable.

**Table 1:** Primer sequences used in gut content analysis.

Amplicon	Primer	Direction	Sequence
rbc	rbcLa_SI_For	Forward	5′-ATGTCACCACAAACAGAGACTAAAGC-3′
rbc	rbcLa_SI_Rev	Reverse	5′-GTAAAATCAAGTCCACCRCG-3′
psb	psbA3'f	Forward	5′-GTTATGCATGAACGTAATGCTC-3′
psb	trnH	Reverse	5′-CGCGCATGGTGGATTCACAATCC-3′
matK	matKfor_KIM3F	Forward	5′-CGTACACAGTACTTTTGTGTTTACGAG-3′
matK	matKrev_KIM1R	Reverse	5′-ACCCAGTCCATCTGAAATCTTGGTTC-3′

PCR reactions were prepared using five µl template, 20 µl GoTaq Green PCR master mix (Promega M7122; Madison, WI, USA), 0.5 µM F and 0.5 µM R primer, and water to a final volume of 40 µl. Thermocycler conditions for *rbcLa* and *psbA-trnH* were: 95 °C for 3 min, 35 cycles × (95 °C 30 s, 55 °C 30 s, 72 °C 1 min), 72 °C for 10 min and for *matK* were: 95 °C for 3 min, 40 cycles × (95 °C 30 s, 50 °C 30 s, 72 °C 1 min), 72 °C for 10 min. A total of 10 µl was run on a 2% agarose gel with exACTGene one kb Plus DNA Ladder (Fisher BP2579-100; Waltham, MA, USA) and assessed for banding pattern. Samples were analyzed with *rbcLa* and *psbA-trnH* primers first, and then PCR was conducted with *matK* primers if needed to achieve a minimum of two successful primer sets per sample. For positive samples, the remaining 30 µl of PCR product was purified using a Wizard PCR Clean-Up System (Promega A9281; Madison, WI, USA) and resuspended in 50 µl nuclease-free water. Two µl of purified PCR product was mixed with 25 ng of the appropriate forward primer for the amplicon (*rbcLa*, rbcLaLa_SI_For; *psbA*, psbAA3'f; *matK*, matKfor_KIM3F) in a 15 µl reaction volume and sequenced using Sanger sequencing through Genewiz (Genewiz, South Plainfield, NJ, USA). Sequences were assessed for plant identity by BLASTing against Genbank sequences at the National Center for Biotechnology Information and assigned to the genus level based on 99–100% identity of the BLAST hit. For a given katydid, we assigned the plant identity of its gut contents when at least two primer sets amplified the same genus. Assignment to the species level was previously found to be 95% accurate with the combination of both *psbA-trnH* and *rbcLa* loci and 92% accurate with the combination of the *matK* and *rbcLa* loci (Kress et al., 2009).

### Statistical analysis

To determine whether the size of the katydid affected sequencing success, we used logistic regression from the base package of R. Mass data were only available for specimens collected in 2017 (*N* = 124). We used Chi-square analyses to test whether sequencing success was affected by the sex of the katydid, the time of day that it was caught, or the color of the gut contents. All analyses were conducted in R (R Development core team, 2013).

## Results

We sampled 238 individuals of 18 species representing a broad range of Phaneropterine katydids (complete data set available in Table S1). From these samples, 48% yielded a product with at least one set of primers. In one species of katydid *(Chloroscirtus discocercus)*, we never recovered plant sequence despite extracting gut contents from 15 individuals. We have removed this species from the analyses below and address it separately in the discussion. In addition, the handling and storage of katydids had substantial impacts on sequencing success. During the course of the project, we attempted DNA extraction and amplification from katydids that had been collected and frozen the previous year. These samples had an extremely high failure rate and are not included in this analysis.

### PCR and sequencing success

**Figure 3:**
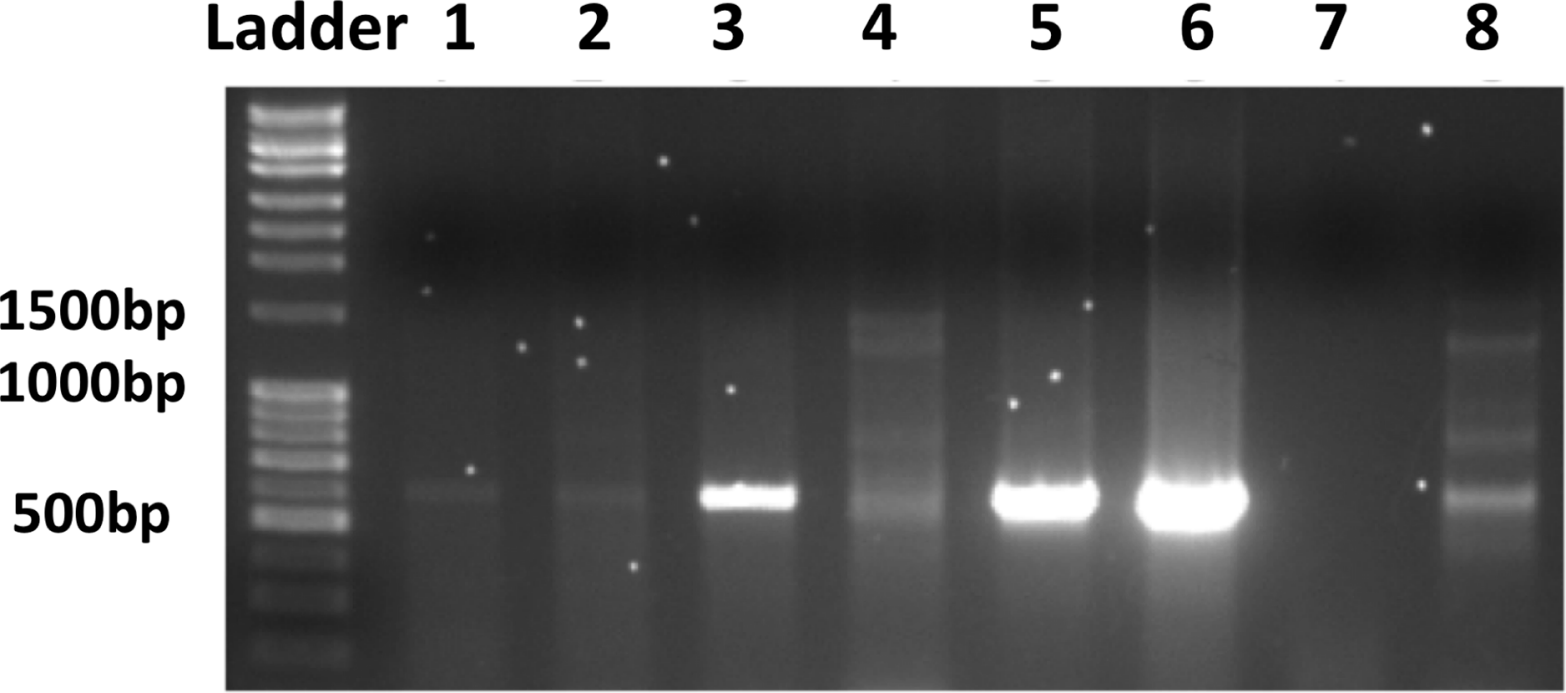
A representative PCR gel showing the range of outcomes. Samples from PCR with *rbcLa* primers were run on a 2% agarose gel and visualized. Reactions generated single strong (lane 3, 5, 6), single weak (lane 1, 2), multi strong (not pictured), multi weak (lane 4, 8) or no amplicon (lane 7).

PCR of gut contents yielded one of five possible outcomes (Fig. 3). The first and optimal outcome was a PCR product with a single strong amplicon. The second outcome was a single weak amplicon, which was characterized by a faint PCR product when visualized on a gel. In a small subset of samples, PCR resulted in multiple amplicons, which appeared on the gel as multiple bright bands (third outcome) or multiple weak bands (fourth outcome). We hypothesize that multiple bands could be a result of the presence of multiple plants in the gut, as plant species can differ in the number of base pairs between the primer binding sites, leading to products of different lengths. The fifth outcome was failed PCR with no product visible on the gel. *rbcLa* primers were the most likely to yield a strong PCR product, and *psbA-trnH* primers were slightly less successful overall (Table 2), consistent with the findings of the researchers that developed these primers (Kress et al., 2009). *matK* primers were utilized to clarify sequencing results or supplement samples that were successful for only *rbcLa* or *psbA-trnH* primers. If neither *rbcLa* nor *psbA-trnH* primers yielded a PCR product for a given sample, *matK* was also unsuccessful on that sample (Table 3). Sequencing success could be anticipated based on the strength of bands on the PCR gel, with strong bands sequencing most successfully (Table 4). Amplicons with single and multiple strong bands sequenced successfully, showing clear chromatograms with single peaks and longer read lengths. Weak amplicons of both single and multiple bands failed to sequence, characterized by very short sequencing reads (<15 bp sequence), even when PCR was repeated on gel-purified weak amplicons. Examples of plant genera that were identified in three katydid species are provided in Table S2.

**Table 2:** PCR outcomes for *rbcLa* and *psbA-trnH* primers.

Outcome	Primer			
	*rbcLa*		*psbA-trnH*	
	Percent	*N*	Percent	*N*
Single strong	35.5	77	19.2	42
Single weak	8.8	19	13.2	29
Multi strong	1.4	3	3.2	7
Multi weak	2.3	5	2.3	5
Failed	52.1	113	62.1	136
Total	100.0	217	100.0	219

**Note:**

Outcome represents the quality and strength of the amplicon band visualized by gel electrophoresis. Percent represents the percentage of samples that displayed a given outcome.

**Table 3:** PCR outcomes for the *matK* primer set.

*rbcLa* outcome	*psbA-trnH* outcome	*matK* outcome	Percent	*N*
Single strong	Single strong	Single strong	31.8	7
		Single weak	40.9	9
		Failed	27.3	6
Single strong	Multi strong	Single strong	25.0	1
		Failed	75.0	3
Single strong	Failed	Single strong	33.3	2
		Single weak	33.3	2
		Failed	33.3	2
Failed	Failed	Single strong	3.6	1
		Single weak	3.6	1
		Failed	92.9	26

**Note:**

Outcomes for *matK* amplification are shown as a function of the PCR outcome for *rbcLa* and *psbA-trnH* primer sets.

**Table 4:** Sequencing outcome based on the visual state of the PCR amplicon on the gel.

Bands on gel	Band condition	Sequencing successful	*N*	% Successful
Single band	Strong	Yes	64	83.1
		No	13	
	Weak	Yes	0	0.0
		No	21	
Multiple bands	Strong	Yes	7	77.8
		No	2	
	Weak	Yes	2	33.3
		No	4	

### Size of insect

**Figure 4:**
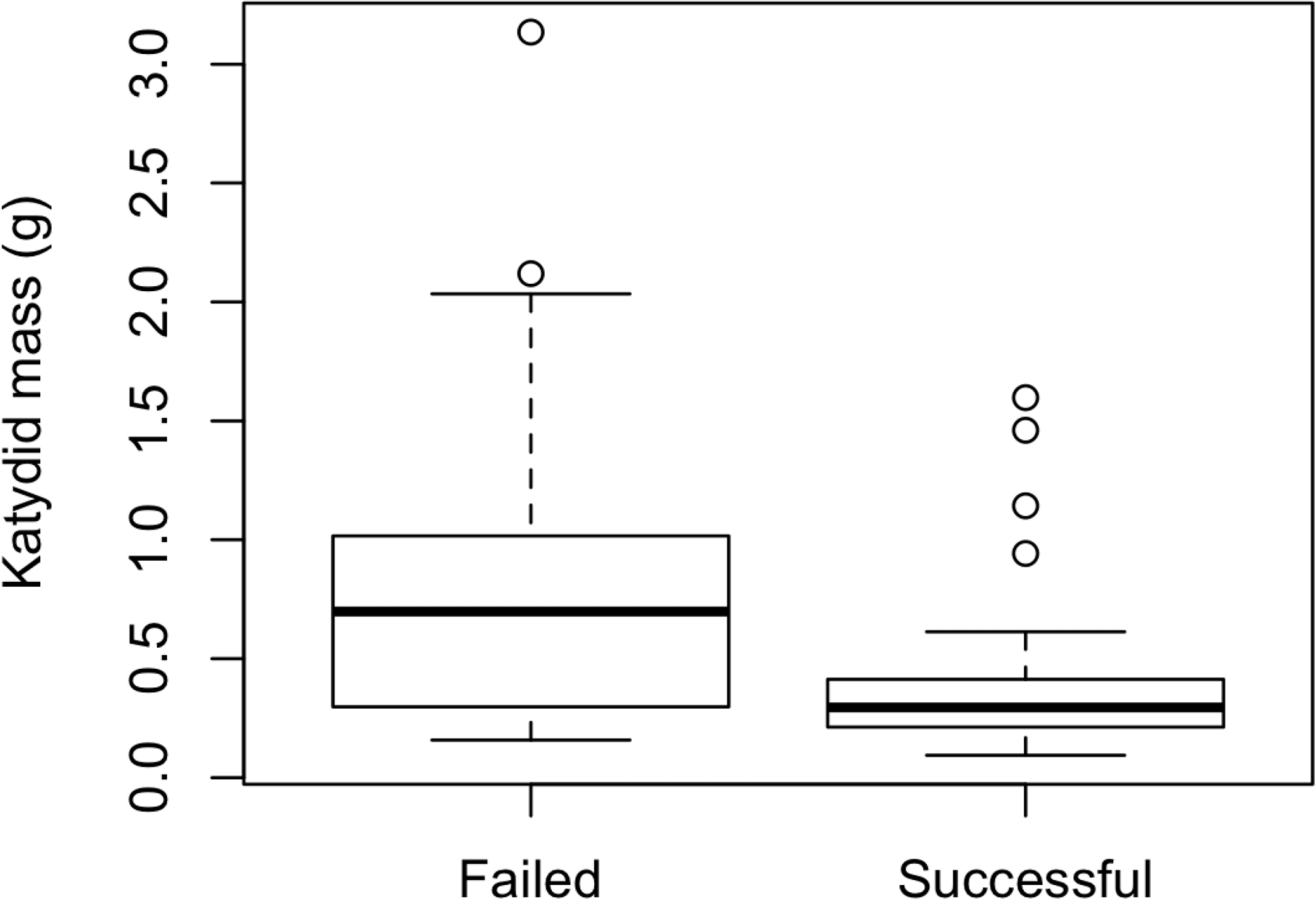
Katydid size and sequencing success. Successful DNA amplification and sequencing was higher in smaller katydids (logistic regression, *p* = 0.04, d*f* = 122).

Gut content sequencing was more successful in small katydids (logistic regression, *p* = 0.04, d*f* = 122, Fig. 4). The median mass of katydids with successfully sequenced gut contents was 0.305 g, compared to a median of 0.517 g in specimens where extraction or sequencing was unsuccessful.

### Sex of insect

Our katydid sample was 65% male, which reflects an underlying male bias in the sex ratio of katydid captures. Gut contents were successfully sequenced from 36% of males and 42% of females. There was no difference between male and female katydids in the probability of success (Chi-square = 0.45, *p* = 0.50).

### Time of capture

Of the katydids that were tested, 45% were captured in the midnight sample (46% of females and 45% of males were caught at the midnight sample). Katydids captured in the midnight sample yielded successful sequences 42% of the time, compared to 32% in the pre-dawn sample (Chi-square = 2.79, *p* = 0.095).

### Color of gut contents

**Figure 5:**
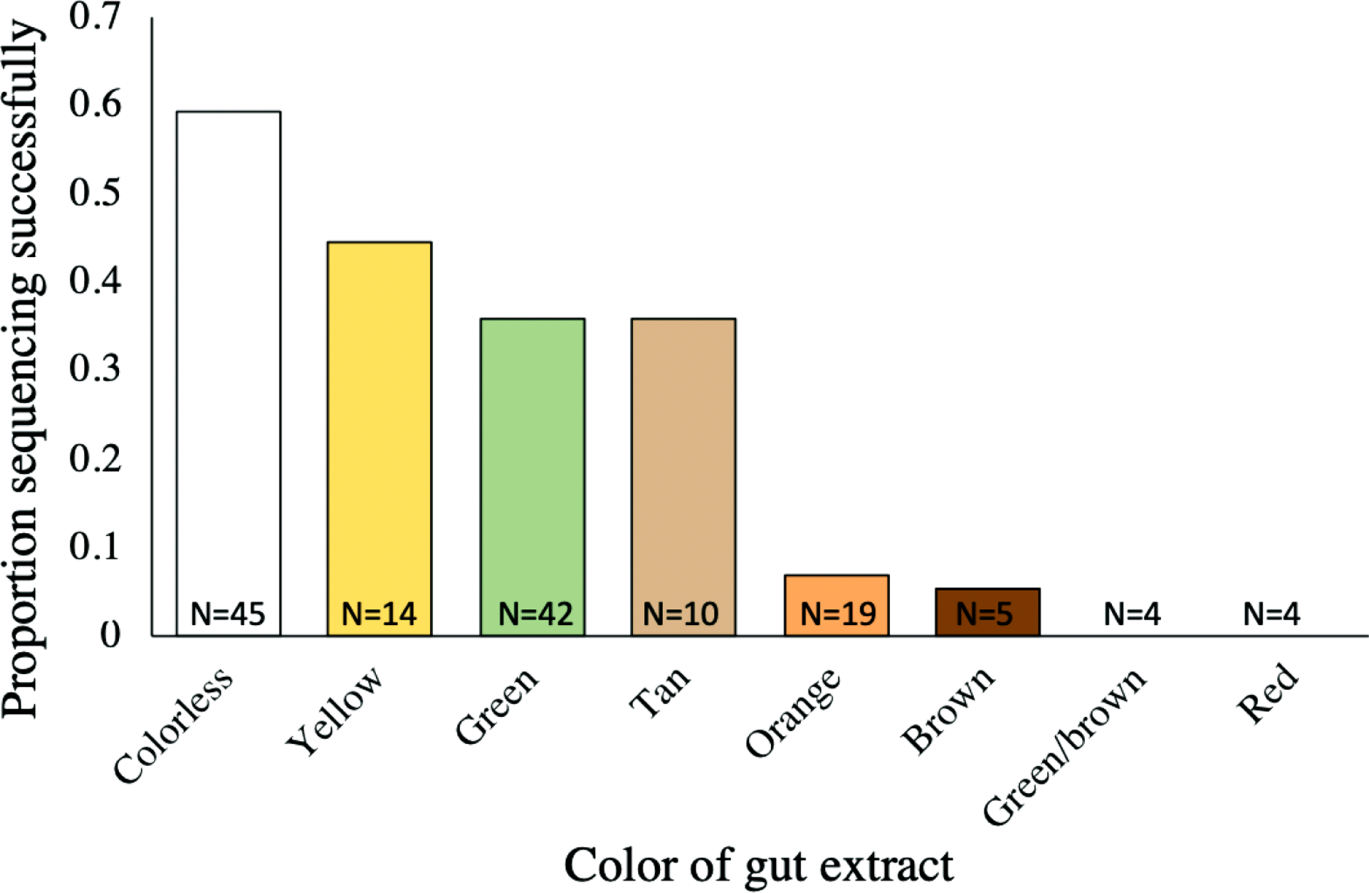
Gut color and proportion of samples with sequencing success. Gut color and proportion of samples with sequencing success. The total number of extracts of a given color is displayed on each bar (Chi-square = 25.96, d*f* = 8, *p* = 0.001).

Gut color was documented for a subset of samples each year (165 of 219 total samples). There were significant differences in sequencing success depending on gut content color (Chi-square = 25.96, d*f* = 8, *p* = 0.001, Fig. S1). When gut contents were colorless, yellow, green, or tan, sequencing was quite successful (>30% success rate), with up to ~60% success when gut contents were colorless (Fig. 5). In contrast, when guts were brown, orange, red, or green–brown, sequencing was rarely or never successful (Fig. 5).

## Discussion

Extracting and sequencing the gut contents of Neotropical forest katydids made it possible to identify plants that are part of their diet (Table S2). To amplify DNA, we employed the three primer sets from Kress et al. (2009) that had been used to identify vegetation on BCI. We found that the primer that targeted the most conserved region (*rbcLa*) had the highest success rate in obtaining PCR product; this is consistent with the results of Kress et al. (2009). While the most conserved primer set, *rbcLa*, yielded a PCR product more than the other primer sets, it also had the lowest specificity. The most specific primer set *(matK)*, which targeted the most variable region, had the lowest PCR success rate. If the *rbcLa* primer set did not yield a product, the *matK* primer set was unlikely to work (Table 3).

Approximately one-third of the sampled katydids ultimately yielded plant DNA that was successfully sequenced. Although this was lower than the rates from direct amplification from plant material (Bafeel et al., 2011; Burgess et al., 2011), the success of gut content amplification was relatively high given the constraints of partial digestion and diversity of leaves, seeds, fruits, and other plant parts that may be consumed. There are a number of insect features that could be associated with sequencing success. Of all of the factors considered, the color of the gut content extract had the strongest association. When the extract was colorless, green, or yellow, the success of extraction and sequencing was relatively high (30–60%). Gut extracts that were brown in color had relatively low PCR success rate and may reflect plant material that was more digested, given that we sometimes observed a green–brown transition from foregut to hindgut (Fig. 2). Extracts that were red were never successful and orange extracts had low success. These red-spectrum colors may be the result of katydids eating fruits, flowers, or new leaves, which in the tropics often lack chlorophyll and are red at first emergence (Kursar & Coley, 1992). These red food sources may have lower levels of plastid DNA or be high in phenolics, including the breakdown products of anthocyanins, and terpenoids, two groups of compounds that bind to DNA and may decrease extraction success, necessitating the use of kits that specifically remove secondary metabolites during extraction (Jobes, Hurley & Thien, 1995). For insect systems with a high prevalence of red gut extracts and low amplification success, we suggest testing extraction protocols that are specifically tolerant to the presence of secondary metabolites and other plant toxins.

Counter-intuitively, gut content sequencing was more successful for smaller katydids. The finding that small katydids are most likely to yield plant DNA that could be amplified suggests that sequencing success is not limited by the volume of gut material available. At the same time, reducing the amount of gut material used from large katydids to the approximate volume we found in the guts of smaller katydids did not enhance sequencing success (C. Palmer, 2018, personal observation), indicating that an overabundance of material is not the limiting factor that is causing low sequencing success in larger katydids. It is possible that small katydids may be feeding more often than larger katydids, or on plant sources that are less likely to contain compounds that negatively affect DNA extraction and sequencing.

While the sex of the katydid did not affect the sequencing success, there was a trend toward higher success in katydids that were collected in the midnight sample. If katydids that are collected during the first half of the night are indeed more likely to provide viable plant sequences, this could reflect either an early evening feeding bout, or the possibility of diurnal feeding. Although most katydids on BCI are thought to be nocturnal, a radio tracking study of the katydid *Philophyllia ingens* found daytime movement toward the canopy, an observation that raised the possibility that at least some species of katydid are active and feeding during the day (Fornoff, Dechmann & Wikelski, 2012). In future studies, it could be useful to compare the plant DNA content of guts from katydids caught at different times of day and night, to determine when feeding occurs in different katydid species.

We were never successful in amplifying plant DNA from the katydid *C. discocercus*, an abundant mid-sized green species. The lack of success is notable because our sample included 15 *C. discocercus*, and based on our success rate with other katydid species, we would expect successful individuals from a pool of this size. The first and most compelling hypothesis is that the intrinsic chemical profile of the *C. discocercus* digestive system is incompatible with intact DNA extraction, whether by altering digestion rates of consumed material or by interfering with the DNA extraction process. A second interpretation is that because our sample included only *C. discocercus* that were captured in the pre-dawn sampling, the failure to recover plant material may reflect unique early evening feeding patterns, leading to insufficient intact plant material in the gut for amplification by the time of capture. This hypothesis is less compelling because material was visible in the digestive tracts, indicating that gut contents were at least present, even if degraded. Other hypotheses for the lack of sequencing success include the possibility that *C. discocercus* is a dietary specialist on either a plant that contains compounds that inhibit DNA extraction, a plant for which the primers used are not effective, or a part of the plant with prohibitively low abundance of plastid DNA. These hypotheses are less compelling because *C. discocercus* digestive tracts were a variety of different colors, suggesting relatively generalized feeding patterns. A final possibility is that in the wild, *C. discocercus* is eating a food source other than plants. While we cannot rule out this possibility, this species does consume and subsist on plant material in the lab.

Taken together, our work provides a guide for field researchers seeking to use a rapid, simple, and resource-efficient method to identify consumed plants in their study system. There are two stages at which the researcher can increase efficiency and reduce costs. First, during insect processing, researchers can assess the color of gut extractions to predict success, with clear/green samples moving forward with a high probability of success. Second, these samples can then be assessed using PCR on-site with user-friendly mini PCR machines and portable electrophoresis units. Any samples that produce a strong band(s) are likely to be successful in sequencing. By assessing the samples at each of these stages, the researcher minimizes wasted time and money by focusing on samples with the highest probability of success, providing quick and field-compatible feedback.

## Conclusions

Identification of consumed plants through DNA extraction and amplification of gut contents provides new insight into the diet of herbivorous insects and is an exciting advance in the understanding of herbivore ecology. We have presented factors that can help the researcher anticipate sequencing success and that will increase efficiency and decrease costs for future investigators. We demonstrate that the highest probability of sequencing success was found in (1) small katydids and (2) gut contents that were yellow, green, or colorless. Katydids that were larger and had red-spectrum gut contents had higher failure rates in DNA amplification. In all cases, the highest sequencing success came from samples that gave a strong gel band following PCR. At each stage, selecting and focusing on the samples with the highest probability of success will be the most efficient and cost-effective approach for quickly and inexpensively identifying plants that a given insect species can consume. If the goal of the research is to exhaustively determine every plant that is present in the diet or to be certain of obtaining diet results for a given individual, then NGS will be a more resource-intensive, but comprehensive approach. Molecular approaches to characterizing diet provide much more detailed resolution of food webs and have the potential to increase our understanding of these Neotropical forest katydids as well as other poorly understood but tropically-important species.

## Supplemental Information

10.7717/peerj.6808/supp-1 Supplemental Information 1 This figure shows representative samples of pulverized gut contents to demonstrate the range of colors included within each color designation.

10.7717/peerj.6808/supp-2 Supplemental Information 2 Original size, sex, and mass data of katydids used for gut content sequencing.

10.7717/peerj.6808/supp-3 Supplemental Information 3 Example genus-level diet data for three of the Phaneropterine species shown in Fig 1 (plant DNA amplification was never successful in the fourth species, *Chloroscirtus discocercus*).

## Additional Information and Declarations

### Data Availability

The following information was supplied regarding data availability: The original size, sex, and mass of katydids used for gut content sequencing is available in Table S1. The original color data are available in Table S2.
