# Supplementary figures and images for: Applying and refining DNA analysis to determine the identity of plant material extracted from the digestive tracts of katydids
Source: PeerJ. 2019 May 3;7:e6808. doi: 10.7717/peerj.6808 (PMC6501762; doi:10.7717/peerj.6808)

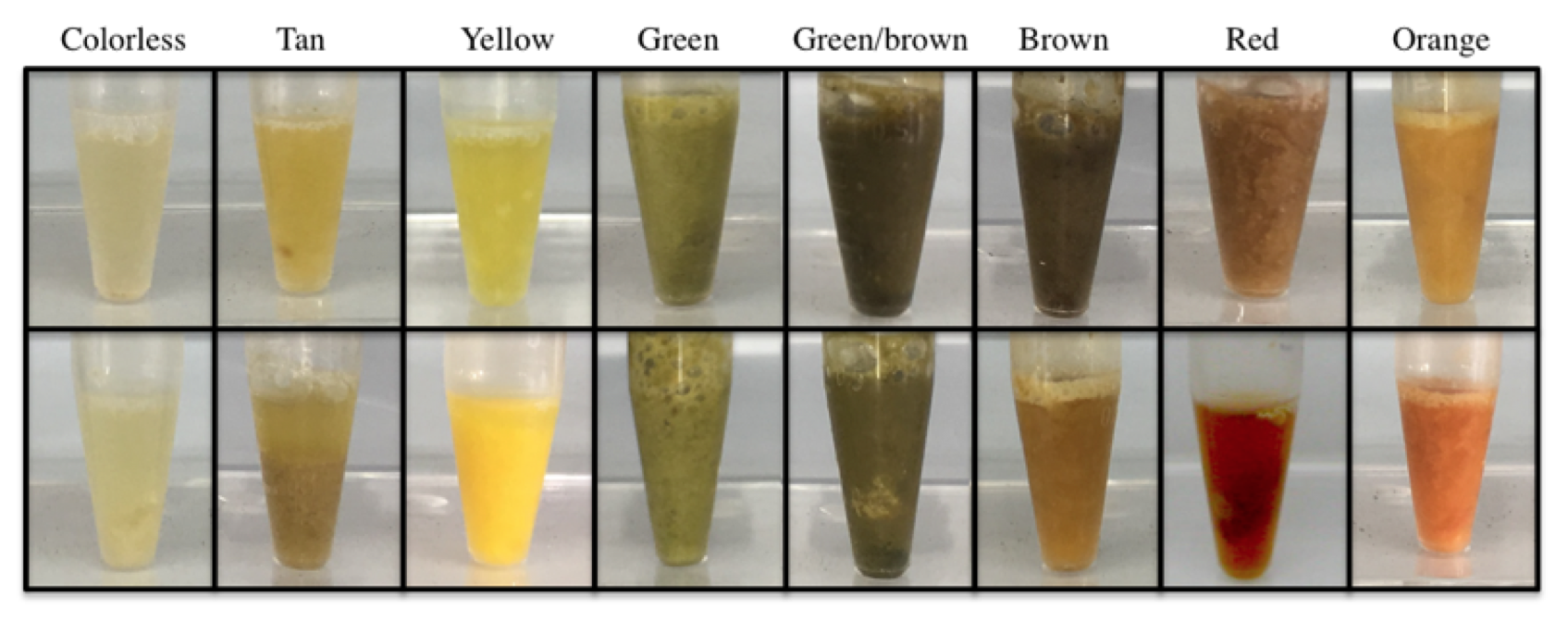

Supplement: Supplemental Information 1 [file peerj-07-6808-s001.png]
