# Supplementary material for: Applying and refining DNA analysis to determine the identity of plant material extracted from the digestive tracts of katydids
Source: PeerJ. 2019 May 3;7:e6808. doi: 10.7717/peerj.6808 (PMC6501762; doi:10.7717/peerj.6808)
Supplement: Supplemental Information 3 [file peerj-07-6808-s003.docx]

**Supplemental Table 2. Sample genus-level diet data for three of the Phaneropterine species shown in Fig 1 (plant DNA amplification was never successful in *Chloroscirtus discocercus*).**
